# Beyond public health genomics: proposals from an international working group

**DOI:** 10.1093/eurpub/cku142

**Published:** 2014-08-27

**Authors:** Stefania Boccia, Martin Mc Kee, Roza Adany, Paolo Boffetta, Hilary Burton, Anne Cambon-Thomsen, Martina C. Cornel, Muir Gray, Anant Jani, Bartha Maria Knoppers, Muin J. Khoury, Eric M. Meslin, Cornelia M. Van Duijn, Paolo Villari, Ron Zimmern, Alfredo Cesario, Anna Puggina, Marco Colotto, Walter Ricciardi

**Affiliations:** ^1^Section of Hygiene-Department of Public Health-Faculty of Medicine, Università Cattolica del Sacro Cuore, Rome, Italy, ^2^Department of Health Services Research and Policy, London School of Hygiene and Tropical Medicine, UK, ^3^Department of Preventive Medicine, Faculty of Public Health, University of Debrecen, Hungary, ^4^Institute for Translational Epidemiology and Tisch Cancer Institute, Icahn School of Medicine at Mount Sinai, New York, NY, USA, ^5^PHG Foundation, Cambridge, UK, ^6^Institut National de la Santé et de la Recherche Médicale and Université Toulouse III Paul Sabatier, Joint Unit 1027 “Epidemiology and analyses in public health”, Faculty of Medicine, Toulouse, France, ^7^Clinical Genetics & EMGO Institute for Health and Care Research, VU University Medical Center, Amsterdam, The Netherlands, ^8^Better ValueHealthCare, UK, ^9^Centre of Genomics and Policy, Faculty of Medicine, Department of Human Genetics, McGill University, Montreal, Canada, ^10^CDC Office of Public Health Genomics, Atlanta, USA, ^11^Indiana University Center for Bioethics, School of Medicine, Indianapolis, USA, ^12^Genetic Epidemiology Unit, Departments of Epidemiology and Clinical Genetics, Erasmus University Medical Center, Rotterdam, Netherlands, ^13^Department of Public Health and Infectious Diseases, “Sapienza “University of Rome, Rome, Italy, ^14^PHG Foundation, Cambridge, UK and ^15^Area of Systems Medicine, IRCCS San Raffaele Pisana, Rome, Italy

Advances in genomics have crucial implications for public health, offering new ways of differentiating individuals and groups within populations that go beyond the measures normally used by public health professionals, such as gender, age, socio-economic status, physiological measurements or clinical biomarkers.^1^ While public health has traditionally been concerned with interventions at a population level, genomic medicine seems to promote a vision for health care that encourages individualism rather than collectivism.^2^ This tension is apparent in weighing up its consequences. Thus, it may bring benefits in stratifying individuals according to genetic risk, enabling better targeting of preventive and therapeutic interventions. But it may also have harmful consequences undermining the imperative to tackle social and environmental determinants of disease and the collective provision of health care potentially leading to overdiagnosis/overtreatment; it may fragment the risk pooling that underpins social solidarity; and it may increase the probability of stigmatization and discrimination.

Consequently, the public health community, with its commitment to equity, must take the opportunity to engage with genomic knowledge, ensuring that it advances the population’s health. These issues were explored in January 2014 at the inaugural meeting of an international working group on ‘Beyond Public Health Genomics’, convening leading experts in genomics, public health, clinical sciences, systems medicine, law and bioethics, from many disciplines and countries, at the Università Cattolica del Sacro Cuore in Rome. Its goal, inspired by the 2005 Bellagio statement on public health genomics, defined as the ‘responsible and effective translation of genome-based discovery into population health,^3^ was to generate high value-based proposals to foster the evidence base for implementing genomic discoveries in public health policy and practice, and to ensure necessary action while accounting for the challenge of needing to fund these workstreams in the current environment of diminishing resources.

The contribution of genomics to health is in three main areas. The first is the ability to go beyond traditional phenotypes using genetic and molecular characteristics as a basis for diagnosis and disease classification. Sequencing and other molecular technologies may facilitate the diagnosis in difficult cases, such as children born with learning disability or developmental delay. Newborn screening programmes enable early diagnosis and treatment for a range of rare inherited metabolic conditions before clinical presentation, thereby improving length and quality of life for affected children. Cancers may now be subdivided into different types according to their genetic or molecular signature, as can diabetes, sudden cardiac death and a range of other conditions. For example, women with *BRCA1* mutations may be identified through genetic testing with a view to offer more intensive preventive options, including mammographic screening, chemotherapy or prophylactic surgery.

The second is to guide treatment, the first emerging area being that of pharmacogenomics. The ability to refine diagnostic categories will allow specific treatments, whether in oncology, cardiology or diabetes care. It may also be possible to differentiate patients according to how they metabolize drugs more accurately tailoring the dosages, or to ascertain those at greatest risk of adverse drug reactions. This has obvious benefits not only for the patient but also the health-care system as a whole because resources will provide only the most effective treatments, thus improving outcomes and reducing waste.

The third is to stratify populations according to disease risk or resistance so as to maximize benefits of prevention programmes, using algorithms computed from both genetic and non-genetic parameters. This approach, sometimes termed genetic profiling, is in its infancy and its utility has yet to be shown, as individuals who carry multiple risk alleles and a high risk of disease will likely constitute only a small percentage of the population.

The participants noted a fourth contribution resulting from the appropriate use of pathogen genomics in infectious disease control, but with the meeting’s focus on human genomics, its contribution was not further discussed.

To date, genomics has had only limited impact on health policy and practice. Khoury *et al.*^4^ have described genomic research as falling on a continuum from the initial discovery (T0) to T1, developing candidate health applications; T2, evaluating its applications and developing evidence-based recommendations; T3, integrating evidence-based recommendations into health care and prevention; and finally T4, assessing health outcomes and population impact. They noted that the vast majority of published research remains in the discovery phase, with a few making it to the early stages of translation. But since that paper was written and published, science and technology have advanced rapidly. Notwithstanding frequent calls to support more translational research and actual implementation of proven genomic interventions, in reality, there have been few efforts to evaluate recent discoveries to see whether they are effective in the real world and, if so, for whom and in which circumstances.^5^

Evans and colleagues have proposed some ways in which the potential of public health genomics might be realized.^6^ Rapid and inexpensive sequencing of genes can identify individuals carrying individually rare mutations that confer substantial predisposition to preventable diseases. Examples include use of *BRCA*1 and *BRCA*2 gene testing for hereditary breast cancer: their combined prevalence is around 0.2–0.3% of the general population, but they confer a >70% lifetime risk for breast and ovarian cancer. Also, the four Lynch-associated genes are present in 0.2% of subjects and confer >80% lifetime risk for colon cancer, with potential risk reduction by colonoscopy and prophylactic aspirin treatment. These are obvious candidates for further evaluation to assess whether there is a case for their wider use, drawing on the experience with some existing neonatal screening programmes, such as that for phenylketonuria, where the prevalence of the genetic disorder is much rarer.^7^ Nevertheless, it is clear that such proposals should be subjected to rigorous evaluation using the screening criteria frameworks usually used for screening programme decisions.

We have already noted that the use of genetic profiling for prevention of common disorders presents major challenges, not least because at present there is little evidence that better knowledge of one’s genetic makeup will lead to behaviour change enhancing health if the underlying risk is not very high.^8^ Worse, it may engender fatalism and anxiety. Other key issues to be addressed include judgements about equity, service effectiveness and prioritization. But, the lack of evidence of utility is not the same as evidence of disutility, and there may in the future be room to refine and integrate genomic information for the prevention of common complex disorders.

So is the incorporation of genomics for prevention and public health use a paradigm shift in public health or a marginal increment? The international working group recognizes that the genetic characteristics of an individual or a population are only one set of factors that will determine the pattern and distribution of health and disease. Most obviously, there is a need to understand gene–environment interactions, exemplified by a recent paper that demonstrated the interaction between a genome-derived risk score and consumption of fried foods in increasing the probability of becoming obese.^9^ However, this is still based on single genes, whereas the obvious next step to tackle complex forms of disease will be to involve multiple genes and environmental factors.

Further, the public health community can integrate this work with contributions from other ‘omics’, including the related fields of epigenomics, proteomics and metabolomics, noting, of course, that all of these factors are embedded within the broader social and economic determinants of health. There is a corresponding need for those working at the molecular level to address the social and economic determinants of public health, and to acknowledge the contribution of existing clinical interventions, the combined effects of which have achieved a decline of up to 50% in cardiovascular deaths in the past three decades in many industrialized countries.

Our proposal is therefore a call for greater engagement by those from public health, with geneticists and scientists to engage with modern genomic and molecular science to develop evidence-based approaches for addressing translational barriers of the kind identified above. Two areas of research were identified as low-hanging fruit:

- Effectiveness and cost-effectiveness research of new genomics-based approaches
- Implementation research that aims to assist the decision-making processes in genomic medicine and its use in disease prevention

Outwith the research agenda, practical solutions to effectively translate research findings within health systems will also be needed. This will mean investment through a separate funding stream to ensure that the fruits of such research are actually acted upon and implemented in the real world of policy makers, clinical practitioners and health service managers, a community with different objectives, reward strategies and culture. Additionally, changes will be needed in the practice of public health that aim to integrate advances in genomics into public health competencies and training, and to establish appropriate policy and ethical frameworks to ensure that genomic technologies and information improve health across all segments of the population.

In conclusion, there may be potential to reduce the burden of disease and improve population health through genomics, but we believe that it will be most successful if it is developed as a natural extension of traditional public health approaches. Achieving total integration of the two will require that practitioners work together. Decision-makers in health and science policy may be reluctant to engage not least because of fear of the financial and organizational implications of innovation. Academics may also have a similar reluctance because of their focus on research and lack of attention to the implications of findings for public policy. Public health professionals have a duty to engage in this agenda. Otherwise, there is a danger that the field will be open for those whose motivations are primarily commercial and incompatible with notions of equity and the promotion of health for all. Horizon 2020 challenges researchers in the field of public health to take public health into the genomics era, in particular through its theme of ‘Piloting personalized medicine into health and care systems’.^10^ The public health and policy communities have no real alternative but to engage; the failure to do so can have only adverse consequences for population health.

## Funding

A.C.T. was partially supported by funds from the European Commission FP7 projects ESGI (European Sequencing and Genotyping Infrastructure, GA 262055), 3Gb-TEST (Introducing diagnostic applications of 3Gb-TEST in human genetics, GA 602269), CAGEKID (Cancer Genomics of the Kidney, GA 241669) EuroTARGET (European collaborative project on TArgeted therapy in Renal cell cancer: GEnetic and Tumour-related biomarkers for response and toxicity, GA 259939). Università Cattolica del Sacro Cuore was supported from Linea D1-2013. E.M.M. was supported by UL1RR025761-01/NIH Indiana Clinical and Translational Sciences Institute; Pierre de Fermat Chaire d’Excellence Program, Midi-Pyrénées Regional Council, France; State Grants to Promote Health Information Technology (Health Information Exchange Challenge Program) US Office of the National Coordination for Health Information, Award No: 90HT0054/01, CFDA. R.Z. and H.B. are funded by the PHG Foundation. The PHG Foundation is the working name of the Foundation for Genomics and Population Health, a charitable company registered in England and Wales: Charity No 118664, Company No 5823194. BMK is funded by Genome Quebec.

*Disclaimer: The opinions by authors of this paper do not reflect the official position of the Centers for Disease Control and Prevention*.
